# A Novel TP53 Gene Mutation Sustains Non-Small Cell Lung Cancer through Mitophagy

**DOI:** 10.3390/cells11223587

**Published:** 2022-11-13

**Authors:** Yuanli Wang, Kah Yong Goh, Zhencheng Chen, Wen Xing Lee, Sze Mun Choy, Jia Xin Fong, Yun Ka Wong, Dongxia Li, Fangrong Hu, Hong-Wen Tang

**Affiliations:** 1 School of Life and Environmental Sciences, Guilin University of Electronic Technology, Guilin 541014, China; 2 Precision Medicine Laboratory, The First People’s Hospital of Qinzhou, Qinzhou 535000, China; 3 Program in Cancer and Stem Cell Biology, Duke-NUS Medical School, 8 College Road, Singapore 169857, Singapore; 4 School of Electronic Engineering and Automation, Guilin University of Electronic Technology, Guilin 541004, China; 5 Division of Cellular & Molecular Research, Humphrey Oei Institute of Cancer Research, National Cancer Centre Singapore, Singapore 169610, Singapore

**Keywords:** TP53, mitophagy, lung cancer, NSCLC

## Abstract

Lung cancer is the leading cause of cancer death in the world. In particular, non-small-cell lung cancer (NSCLC) represents the majority of the lung cancer population. Advances in DNA sequencing technologies have significantly contributed to revealing the roles, functions and mechanisms of gene mutations. However, the driver mutations that cause cancers and their pathologies remain to be explored. Here, we performed next-generation sequencing (NGS) on tumor tissues isolated from 314 Chinese NSCLC patients and established the mutational landscape in NSCLC. Among 656 mutations, we identified TP53-p.Glu358Val as a driver mutation in lung cancer and found that it activates mitophagy to sustain cancer cell growth. In support of this finding, mice subcutaneously implanted with NSCLC cells expressing TP53-p.Glu358Val developed larger tumors compared to wild-type cells. The pharmaceutical inhibition of autophagy/mitophagy selectively suppresses the cell proliferation of TP53-null or TP53-p.Glu358Val-expressing lung cancer cells. Together, our study characterizes a new TP53 mutation identified from Chinese lung cancer patients and uncovers its roles in regulating mitophagy, providing a new insight into NSCLC treatment.

## 1. Introduction

According to Global Cancer Statistics (GLOBOCAN) 2020, lung cancer ranked as the second most common cancer and is the leading cause of cancer death worldwide [1]. Non-small cell lung cancer (NSCLC) accounts for approximately 85% of lung cancers whereas the rest are categorized as small cell lung cancer [2]. The advent of high-throughput sequencing technologies accelerates the understanding of the genetic mutation landscape in lung cancers. In particular, the driver mutations in NSCLC now serve as prognostic and predictive biomarkers for treatment options [3,4,5].

Abnormal cell proliferation is one of the hallmarks of cancers. Genetic alterations can promote cell proliferation through dysregulating essential signaling pathways. In NSCLC, KRAS mediates signaling pathways, such as the mitogen-activated protein kinase (MAPK) pathway; RAS-rapidly accelerated fibrosarcoma (RAF)-MAPK extracellular signal-regulated kinase (ERK) kinase (MEK)-ERK pathway; and the phosphatidylinositol 3-kinase (PI3K)-protein kinase B (AKT)-mechanistic target of rapamycin (mTOR) pathway, to control cell proliferation [6]. KRAS-mutant NSCLC exhibits uncontrollable cell growth through interacting with downstream effectors, such as TGF-β1 or IL-10, and contributes to tumorigenesis [7]. Similarly, epidermal growth factor receptor (EGFR) mutations are frequently observed in NSCLC [8]. NSCLC patients with EGFR mutations show an increased cell proliferation, migration and survival through the aberrant activation of the PI3K-AKT pathway, the STAT pathway, and the MAPK pathway [9].

The *TP53* gene is one of the most frequently mutated genes in cancer and is canonically known as a tumor suppressor. In fact, half of NSCLC contains the mutated TP53 gene [10]. TP53 mutants promote tumor growth by activating aberrant cell proliferation. For instance, concomitant TP53 mutations in NSCLC activate the aforementioned intracellular pathways that involve EGFR translocation and also worsen the KRAS-driven oncogenic properties [11]. Furthermore, TP53 mutations accompanied by an increased autophagy can accelerate tumor growth [12]. Autophagy is an important molecular degradation mechanism that maintains cellular homeostasis [13]. During autophagy, double-membrane vesicles, called autophagosomes, engulf organelles or proteins and subsequently fuse with lysosomes to degrade the contents within the autolysosomes [14]. This process is strictly regulated by multiple autophagic regulators such as mTORC1, TRAF6, AMPK, ZIPK, TFEB, FOXO, Dwg, and P53 [15,16,17,18,19,20,21]. Autophagy has been reported to play different roles during various stages of tumor development. Before tumor formation, autophagy inhibits abnormal cell growth and thereby suppresses tumorigenesis. However, after the tumor develops, autophagy, in turn, promotes cancer cell survival in harsh microenvironments [22,23,24]. The upregulation of autophagy by TP53 can further confer tumor cell resistance towards radiation therapy, chemotherapy and targeted therapy [25], highlighting the roles of autophagy in TP53-related tumors.

Although multiple studies have linked TP53 to autophagy, the regulation of autophagy by TP53 remains debatable. Oncogenic activation triggers a TP53-dependent autophagy that relies on nuclear TP53 transcriptional activity [26]. Interestingly, another study by Tasdemir et al. has reported that endogenous TP53 could repress macro-autophagy via the inhibition of AMPK and the activation of mTOR [27]. Furthermore, a recent study reported that TP53 suppresses mitophagy, a selective form of autophagy that eliminates dysfunctional or damaged mitochondria. TP53 inhibits the transcription of Pten-induced kinase 1 (PINK1), the sensor of mitochondrial damage, and thus suppresses PINK1-mediated mitophagy [28]. These studies suggest the duality in the ability of TP53 to control autophagy. The regulation of autophagy by TP53 may depend on its subcellular localization, mutation status, tissue type or the level of stress.

By characterizing gene mutations in Chinese lung cancer patients, we identified TP53- p.Glu358Val as a loss-of-function mutation in lung cancer and found that it activates mitophagy to increase cancer cell growth. The pharmaceutical inhibition of mitophagy selectively suppresses the cell proliferation of TP53-null or TP53-p.Glu358Val-expressing lung cancer cells. Together, our study characterizes a new TP53 mutation and uncovers its roles in regulating mitophagy, laying a foundation for the development of new NSCLC therapies.

## 2. Materials and Methods

### 2.1. NSCLC Patients

The 314 patients in this study were diagnosed with NSCLC (non-small cell lung cancer) and underwent surgeries or biopsies at the First People’s Hospital of Qinzhou between October 2020 and October 2021. Clinical and pathological information were obtained from medical records, including age, clinical and pathological stages, histological types and treatments. Lung cancer tumor tissues were collected from the patients, immediately snap-frozen and kept at −80 °C until DNA extraction.

### 2.2. Next-Generation Sequencing (NGS)

DNA in lung cancer tissues was extracted by following the total nucleic acid extraction protocol of the QIAamp kit (Qiagen) and was subjected to next-generation sequencing (NGS). DNA samples were sequenced on Illumina Miniseq platforms (27-gene panel; EGFR, TP53, KRAS, SMD4, ALK, PIK3CA, BRAF, STK11, CTNNB1, PTEN, ERBB2, FGFR2, MET, FGFR1, NRAS, ERBB4, FBXW7, FGFR3, NOTCH1, ROS1, RET, NTRK1, MAP2K1, DDR2, AKT1, NTRK2 and NTRK3).

### 2.3. Mutation Count

For quantification of mutation spectrum, the mutation count is calculated by expressing individual gene mutation counts over the sum of total gene mutation counts.

### 2.4. Cell Lines and Culture Conditions

Human NSCLC cell line (NCI-H2172) was grown in RPMI 1640 (Invitrogen, Waltham, MA, USA) supplemented with 10% fetal bovine serum (FBS) and 1% streptomycin–penicillin (Sigma-Aldrich/Merck, St. Louis, MO, USA). NCI-H2172 and HEK293T cells were gifts from Prof. David Virshup (CSCB Department, Duke-NUS Medical School, Singapore). HEK293T cells were grown in Dulbecco’s modified Eagle medium (DMEM), 10% fetal bovine serum (FBS), 1% streptomycin–penicillin (Sigma-Aldrich/Merck), 1 mM sodium pyruvate (Life Technologies, Carlsbad, CA, USA) and 2 mM L-glutamine (Sigma-Aldrich/Merck). Cells were maintained in a humified incubator at 37 °C under 5% CO_2_ atmosphere. All human cell lines were authenticated using STR profiling. All experiments were performed with mycoplasma-free cells.

### 2.5. Lentiviral Vectors Production

HEK293T cells were used to produce lentiviral vectors. A total of 6 × 10^5^ HEK293T cells were seeded into 6-well plate. After 24 h, HEK293T cells were transfected with viral packaging (psPAX2), viral envelope (pMD2G) and plasmids pLX313-TP53-WT (gift from Addgene, #118014) or pLX313-TP53-p.Glu358Val with Lipofectamine 3000 (Invitrogen). pLX313-TP53-p.Glu358Val was generated using PCR mutagenesis by replacing the conserved TP53 Glutamic Acid 358 with Valine. After transfection, lentiviral supernatants were collected, centrifuged and filtered. The viral supernatants were supplemented with 10 µg/mL polybrene and incubated with H2172 cells for 48 h. The lentivirus-infected H2172 cells were selected and maintained by hygromycin B (500 µg/mL). The primer sequences used for TP53-p.Glu358Val PCR mutagenesis are as below. TP53-p.Glu358Val-forward: 5′-GGCTGGGAAGGTGCCAGGGGGGA-3′ and TP53-p.Glu358Val-reverse: 5′-TCCCCCCTGGCACCTTCCCAGCC-3′.

### 2.6. Colony Formation Assay

A total of 1000 cells were plated in six-well plate, with medium being replaced every 3–4 days. After 12 days, colonies were fixed with cold methanol, washed with PBS and stained with 1% crystal violet. Images were taken with GelCount (Oxford Optronix, Abingdon, UK). Crystal violet was extracted by washing with PBST (PBS with 0.1% Triton-X) for 30 min at room temperature and measured with spectrophotometer at wavelength of 570 nm.

### 2.7. The 3-MA Treatment on Cell Viability

H2172, H2172-expressing TP53-WT or H2172-expressing TP53-p.Glu358Val cells were cultured either with or without 5 mmol/L 3-MA for 24 h. Cell viability was measured using the CellTiter-Glo^®^ Luminescent Cell Viability Assay (Promega, Madison, WI, USA) or determined by Trypan Blue exclusion assay.

### 2.8. Bafilomycin A1 Treatment on Autophagy

H2172, H2172-expressing TP53-WT or H2172-expressing TP53-p.Glu358Val cells were cultured in RPMI 1640 media either with DMSO or 0.1 µM Bafilomycin A1 for 24 h. The cells were then subjected to immunofluorescence and Western blotting analysis.

### 2.9. Immunofluorescence

Cells were fixed with cold methanol for 15 min, washed with PBST, blocked with 10% serum for 1 h at room temperature, and then incubated with rabbit polyclonal anti-LC3B (1:200) (2775, Cell Signaling Technology, Danvers, MA, USA) at 4 °C overnight. The anti-rabbit-Alexa Fluor^®^ 488 (Life Technologies, A-11008) was used as secondary antibody at 1:500 dilution for 1 h at room temperature, followed by the addition of DAPI (1 µg/mL) for 10 min. Samples were visualized by Zeiss LSM 710 confocal microscope (ZEISS, Oberkochen, Germany).

### 2.10. Immunoblot Analysis

Cells were harvested and resuspended in RIPA buffer containing protease and phosphatase inhibitors. Protein lysates from each sample were separated by SDS-PAGE and transferred to PVDF membranes. The membranes were blocked and probed with primary antibodies, including anti-PINK1 (Abcam, ab23707), anti-ATP5A (Abcam, ab14748), anti-Tubulin (Merck/Sigma, T5168), anti-p53 (Cell Signaling Technology, 9282) and anti-LC3B (Cell Signaling Technology, 2775) antibodies.

### 2.11. Quantification of mRNA Expression

Total RNA was extracted using TRIzol (Invitrogen 15596-018) following the manufacturer’s instructions, and 500 ng of total RNA was used to generate cDNA with PureNA First Strand cDNA Synthesis Kit (KR01-100, Research Instruments, Singapore, Singapore). Quantitative PCR was then performed using BlitzAmp Hotstart qPCR Master Mix (1204201, Mirxes, Singapore, Singapore) in CFX96 Real-Time System (BIO-RAD, Hercules, CA, USA). All assays were performed in triplicate. The primer sequences used for qPCR are: PINK1 forward-5′-CGAGGAACTCGTTTGAAGGG-3′, PINK1 reverse-5′-CCAGGTGGCAAATCAGACATG-3′, GAPDH forward-5′-CATCTTCCAGGAGCGAGATC-3′, GAPDH reverse-5′-GTTCACACCCATGACGAACAT-3′.

### 2.12. Mouse Xenograft Model

NOD/SCID mice (6–8 weeks old) were subcutaneously implanted with 2 × 10^6^ cells in 100 µL PBS/Matrigel (*v*/*v*, 1:1). Tumors were measured and tumor volumes were calculated with the formula V = 0.5ab^2^ (where a = largest diameter; b = smallest diameter). When tumor volume reached approximately 1500 mm^3^, all of the mice were sacrificed. The tumors were collected for further biochemical analysis. Animal protocol was approved by the Institutional Animal Care and Use Committee of the Duke-NUS Medical School at Singapore.

### 2.13. Statistical Analysis

Results are expressed as mean ± SEM. Student’s *t*-test (two sample comparisons) or one-way ANOVA (multiple sample comparisons) analysis was performed with GraphPad Prism to determine the significance.

## 3. Results

### 3.1. Mutational Landscape of Chinese Lung Cancer

To systematically identify driver mutations in non-small cell lung cancer (NSCLC), we collected NSCLC samples from 314 patients who underwent surgeries or biopsies at the First People’s Hospital of Qinzhou between October 2020 and October 2021. The patients’ age ranged from 29 years old to 94 years old, with the median age of the cohort being 65 years old. Out of 314 patients, 35.67% (112/314) were female and 64.33% (202/314) were male. A total of 86.31% (271/314) of these patients had clinical stage IV lung cancer whereas 4.46% (14/314) of these patients had cancer where its clinical stages were unable to be identified. The patient cohort was further categorized by the TNM system, where T describes the size and extent of the tumor, N refers to the number of affected lymph nodes with cancer and M refers to metastasis. A significant number of patients’ cancer was not able to be measured according to the T, N and M stage. Specifically, 68.47% (215/314) failed to report for the T stage, 67.84% (213/314) for the N stage and 68.79% (216/314) for the M stage. The T4 stage was found in 15.29% (48/314), 16.56% (52/314) presented with the N3 stage and 24.84% (78/314) with the M1 stage. The detailed clinicopathological features of the cohort were summarized in Table 1.

**Figure 1:**
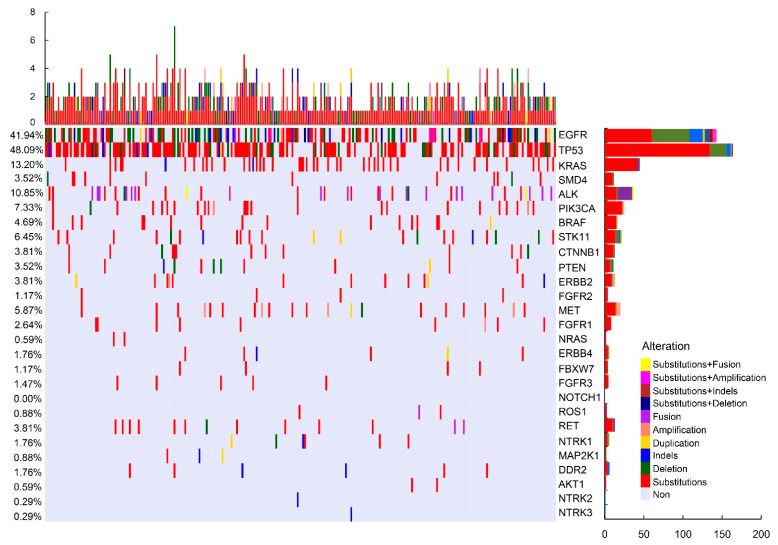
Mutation profile of the cohort. Each row represents a gene and each column represents a patient. The bar located at the bottom left denotes the mutation count of respective genes. Top plot represents the overall number of mutations that a patient carried. Different colors denote different types of mutations.

To delineate the mutational landscape of lung cancers, these collected NSCLC tumor tissues were subjected to Illumina Miniseq platforms (27-gene panel; mutations that include substitutions, deletions, insertion–deletions (Indels), duplication, amplification and fusion of the following genes: EGFR, TP53, KRAS, SMD4, ALK, PIK3CA, BRAF, STK11, CTNNB1, PTEN, ERBB2, FGFR2, MET, FGFR1, NRAS, ERBB4, FBXW7, FGFR3, NOTCH1, ROS1, RET, NTRK1, MAP2K, DDR2, AKT1, NTRK2 and NTRK3). In total, we detected 656 mutations, including 467 substitutions, 94 copy number deletions, 39 insertion–deletion variants (Indels), 39 copy number amplifications (CNA), 14 duplications and 3 fusions, across 27 genes. Our sequencing results revealed that TP53 is the most commonly mutated gene, representing 48.09% of the overall mutations. Some of the commonly known oncogenes, such as EGFR (41.94%), KRAS (13.20%) and ALK (10.85%), were also at the top of the list of mutated genes in the cohort (Figure 1). For TP53 in particular, 83.3% (150/180) of mutations in TP53 are due to substitutions (Figure 1). The detailed distribution of the genomic alterations detected in the lung cancer cohort is summarized in Figure 1 (Figure 1). As observed, not all gene mutations correlate with patient demographics. Notably, MET mutation is significantly associated with age. Patients with EGFR, ALK and KRAS mutations are more likely to be influenced by their gender. N stage cancer patients are significantly less inclined to harbor BRAF, ROS1 and AKT1 mutations (Figure S1).

### 3.2. Classification of the Mutations Found in NSCLC

**Figure 2:**
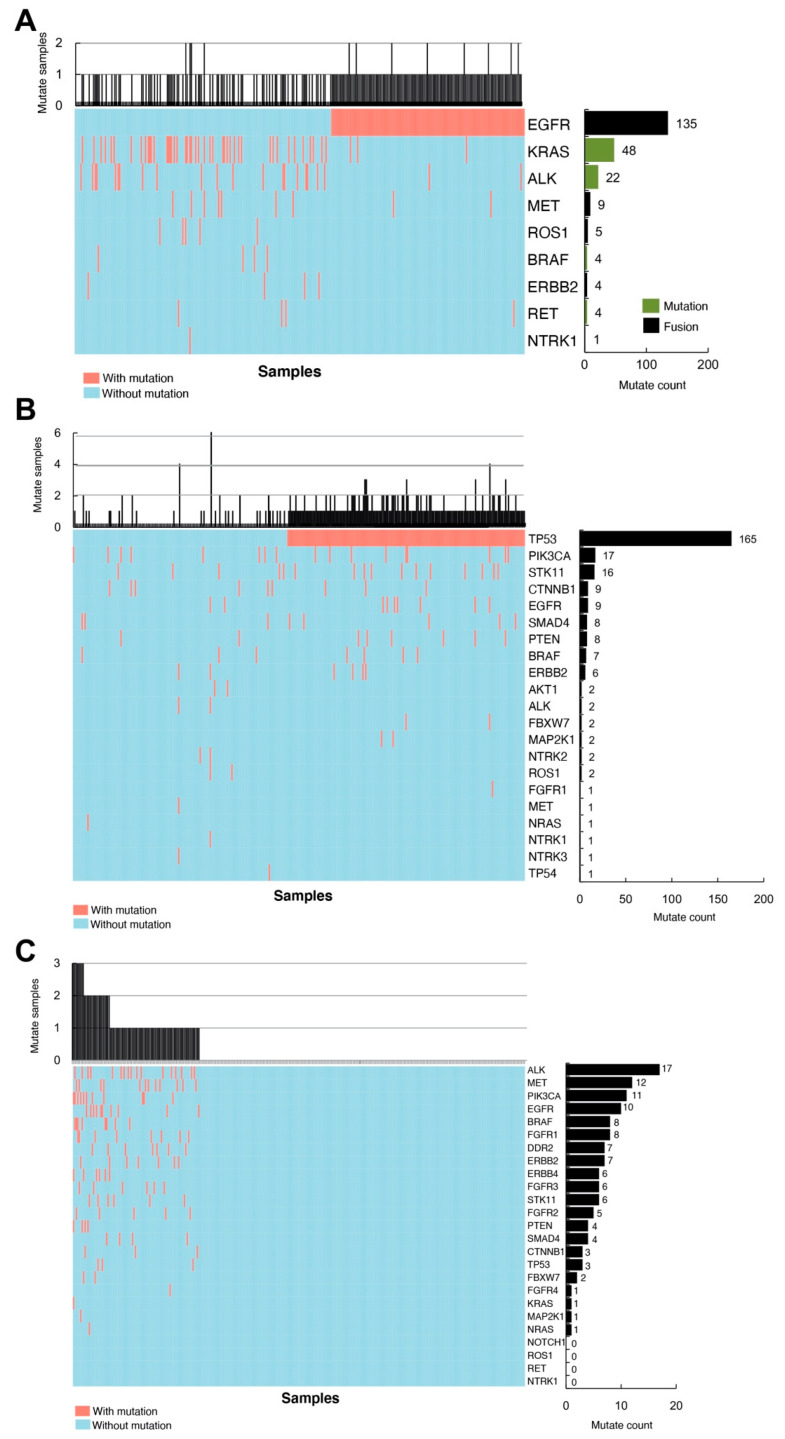
Mutation spectrum of different classes in Chinese lung cancer patients. (**A**) Class I mutations. EGFR has the highest mutation counts due to fusion in Class I. The black bar indicates mutations caused by gene fusion, and the green bar represents mutations due to other forms. (**B**) Class II mutations. TP53 mutations represent the highest count under Class II. (**C**) Class III mutations. ALK gene alteration is most frequently observed in this class.

To classify the identified 656 mutations in NSCLC patients, we first grouped the mutations into three categories based on their functional attributes and drug responses. Class I mutations refer to gene mutations with well-defined effects and approved drug therapies, whereas Class II mutations are gene mutations with known malfunctions but no specific treatments targeting mutation-induced effects. The class III mutation is a group of mutated sites with neither characterization nor known treatments. Upon quantifying mutation counts of each gene out of all of the mutations in each class, we found that EGFR has the highest mutation count (135/232) in class I due to fusion with itself or other mutated genes. Meanwhile, a 20.7% (48/232) mutation count in KRAS was detected under class I in our cohort (Figure 2A). In class II, the most common mutation was TP53, which occupies 62.7% (165/263) (Figure 2B). In class III, the mutated genes were more equally distributed. ALK (17/123), MET (12/123), PIK3CA (11/123) and EGFR (10/123) were more frequently detected under class III (Figure 2C). Interestingly, EGFR and TP53 have the highest number of combinations with other mutated genes in class I and class II, respectively. In class III, PI3KCA and BRAF are the top two mutated genes that combined most frequently with other mutants (Figure S2).

### 3.3. A Loss of Function Mutation, TP53-p.Glu358Val, Increases Lung Cancer Cells Growth

**Figure 3:**
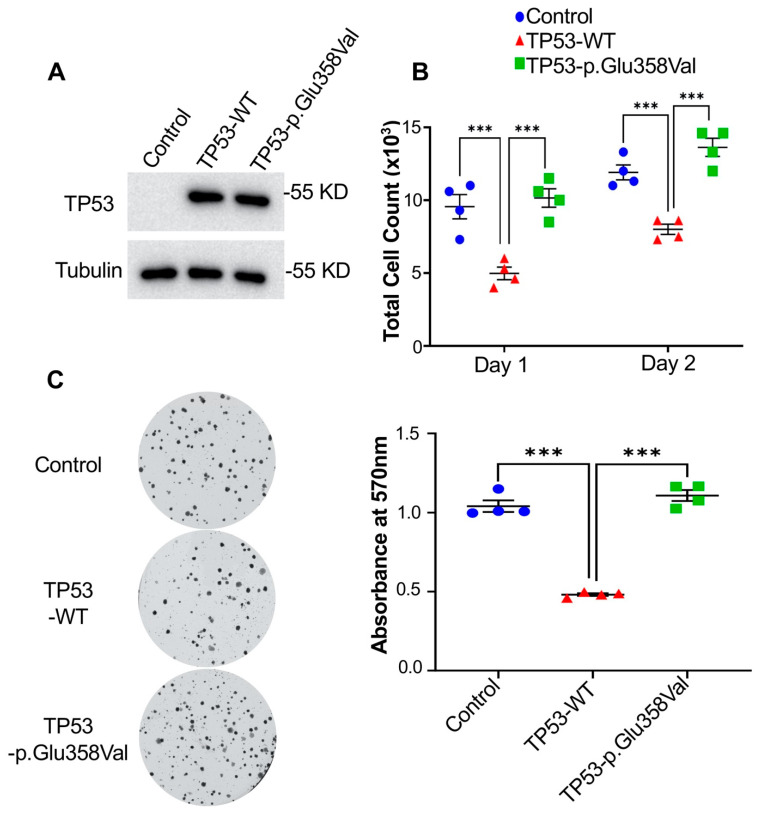
TP53-p.Glu358Val-expressing H2172 cells show increased cell proliferation. (**A**) Immunoblot of TP53 and tubulin proteins in H2172 cells. (**B**) Total cell counts on day 1 and day 2 after seeding. (**C**) Colony formation assay and quantification of crystal violet at 570 nm. *** *p* < 0.001.

To identify new driver mutations in NSCLC, we first tested whether the expression of mutated genes in class III can affect cell growth or proliferation in H2172 cells, a human NSCLC cell line. We expressed multiple genes with mutations in class III and identified TP53-p.Glu358Val as a potential mutation that enhances tumorigenesis. H2172 cells is a human NSCLC cell line that does not express TP53 (TP53 null cells) [29]. Our Western blot revealed that the protein expression levels of wild-type TP53 and TP53-p. Glu358Val are the same in H2172 cells (Figure 3A). In H2172 expressing TP53, cell numbers were reduced by around 50% compared to H2172 cells (Figure 3B). We did not observe significant cell death in H2172 cells expressing wild-type TP53 (data not shown), suggesting that the decrease in the cell number was due to a reduction in proliferation. Surprisingly, the expression of TP53-p.Glu358Val dramatically increases cell proliferation compared to wild-type TP53 (Figure 3B). It is worth noting that the cells expressing TP53-p.Glu358Val mutants grew almost equally or even faster than control H2172 cells (TP53 null cells) (Figure 3B). These results strongly suggest that TP53-p.Glu358Val mutation is a loss-of-function mutation that abrogates the wild-type TP53 function in suppressing cell proliferation.

Consistent with previous results, H2172 cells expressing TP53-p.Glu358Val possessed the same colony-forming capability as control TP53 null cells. However, the colony-forming capability was impaired in cells expressing wild-type TP53 (Figure 3C). As presented in Figure 3C, the quantification of the crystal violet washout of colonies expressing wild-type TP53 at an absorbance of 570 nm was significantly lower than that of H2172 TP53 null control or H2172 expressing TP53-p.Glu358Val cells. Together, our findings demonstrate that TP53-p.Glu358Val mutation abolishes the tumor-suppressive function of TP53 and therefore enhances the growth of lung cancer cells.

### 3.4. TP53-p.Glu358Val Mutation Results in Aggressive Xenograft Tumors Growth

**Figure 4:**
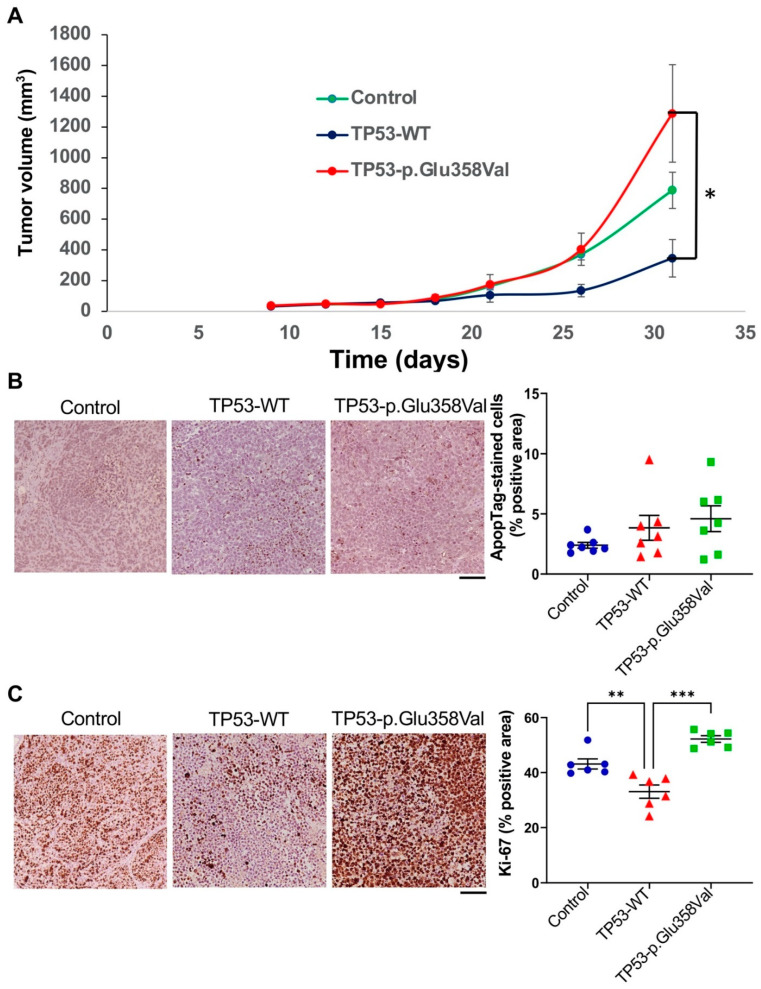
Tumor grafts in mouse model. (**A**) The tumor volume was measured for four weeks. The diagram shows the tumor growth curve of the mouse at the indicated days after subcutaneous implant of the tumor cells (*n* = 5 per group). (**B**) H2172 tumors were sectioned and stained by ApopTag. (**C**) Ki-67 staining of H2172 tumor sections. Scale bar = 200 µm. * *p* < 0.05, ** *p* < 0.01, *** *p* < 0.001.

To further investigate whether TP53-p.Glu358Val mutation contributes to tumorigenesis in vivo, we implanted 2 × 10^6^ cells of H2172 expressing TP53-p.Glu358Val, H2172 expressing TP53-WT and an H2172 TP53 null control subcutaneously in the left and right flanks of mice, and monitored the growth of these xenograft tumors. Consistent with previous in vitro results, the growth rate of xenograft tumors formed by cells expressing TP53-p.Glu358Val was significantly faster than that of TP53-WT-expressing cells (Figure 4A). In addition, our ApopTag assay suggests that no significant cell death was observed (Figure 4B). Immunohistochemical analysis of the nuclear antigen Ki-67 further revealed that the tumors from H2172 expressing TP53-p.Glu358Val exhibit a higher Ki-67-positive area than cells with or without the expression of wild-type TP53 (Figure 4C). These results demonstrate that TP53-p.Glu358Val, a loss of the function mutation of TP53, promotes tumor growth in vivo.

### 3.5. Autophagy Is Required for the TP53-p.Glu358Val-Driven Cell Growth

**Figure 5:**
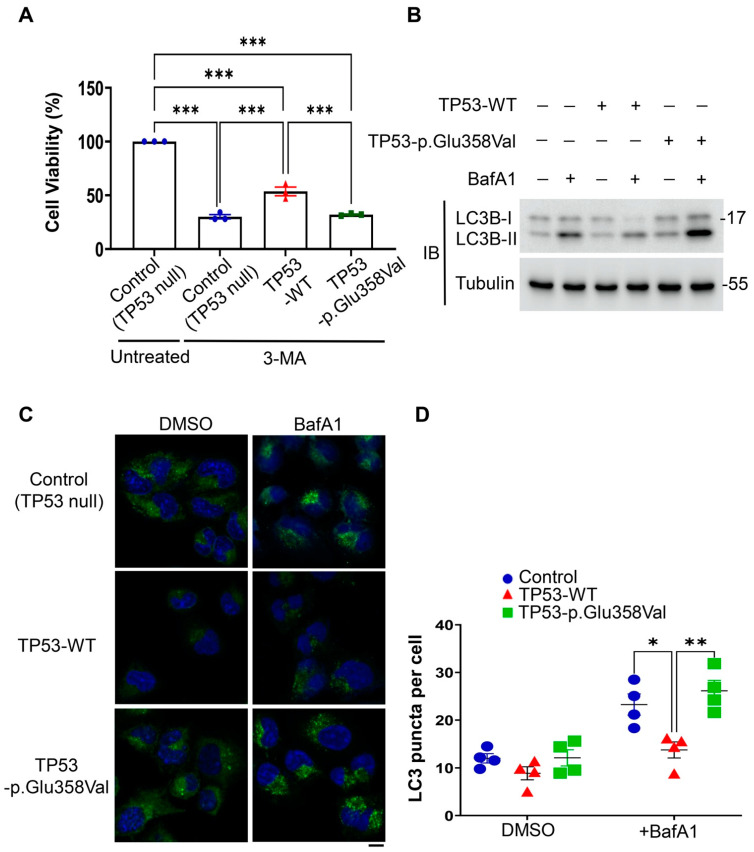
TP53-p.Glu358Val-expressing H2172 cells activate autophagy to sustain NSCLC. (**A**) Cell viabilities after 3-MA treatment. (**B**) Immunoblots of LC3B and tubulin proteins. (**C**) Immunofluorescence of LC3B punctate with or without BafA1 treatment. (**D**) Quantification of LC3 puncta per cell. Scale bar = 10 µm. * *p* < 0.05, ** *p* < 0.01, *** *p* < 0.001.

To explore the mechanism underlying the TP53-p.Glu358Val-driven cell proliferation, we performed a small-scale drug screen on H2172, H2172-expressing TP53-WT and H2172-expressing TP53-p.Glu358Val cells. Interestingly, among candidate drugs, we found that the treatment of 3-methyladenine (3-MA), a class III PI3K inhibitor that suppresses autophagy/mitophagy [30], significantly decreases the viabilities of H2172 TP53 null and H2172 expressing TP53-p.Glu358Val (Figure 5A). In contrast, 3-MA on wild-type TP53-expressing H2172 cells has less of an effect on viabilities (Figure 5A). These results suggest that TP53-p.Glu358Val mutation enhances the dependence of lung cancer cells on autophagy.

To further examine the roles of autophagy in TP53-p.Glu358Val-driven cell proliferation, we examined the level of LC3, an autophagic indicator, in H2172 cells stably expressing wild-type TP53 or TP53-p.Glu358Val in the presence or absence of the lysosomal inhibitor bafilomycin A1 (BafA1). BafA1 inhibits the fusion of autophagosomes with lysosomes, thereby suppressing the autophagosomal degradation. We found that wild-type TP53 expression in H2172 reduced the numbers of LC3 puncta and suppressed the conversion of cytosolic LC3 (LC3-I) to the lipidated form of LC3 (LC3-II), as revealed by the LC3 conversion assay with or without the treatment of BafA1, suggesting that TP53 inhibits the early stage of autophagy in lung cancer cells (Figure 5B–D). On the contrary, TP53-p.Glu358Val expression failed to repress autophagy. H2172 cells stably expressing TP53-p.Glu358Val exhibit increases in the numbers of LC3 puncta and the lipidated LC3 compared to cells expressing wild-type TP53 (Figure 5B–D). Together, our results show that TP53-p.Glu358Val mutant proteins activate autophagy to sustain lung cancer cells survival and cell growth, and that the inhibition of autophagy can selectively inhibit the proliferation of lung cancer cells driven by the loss of function of TP53.

### 3.6. TP53-p.Glu358Val Induces Mitophagy via Up-Regulating PINK1

**Figure 6:**
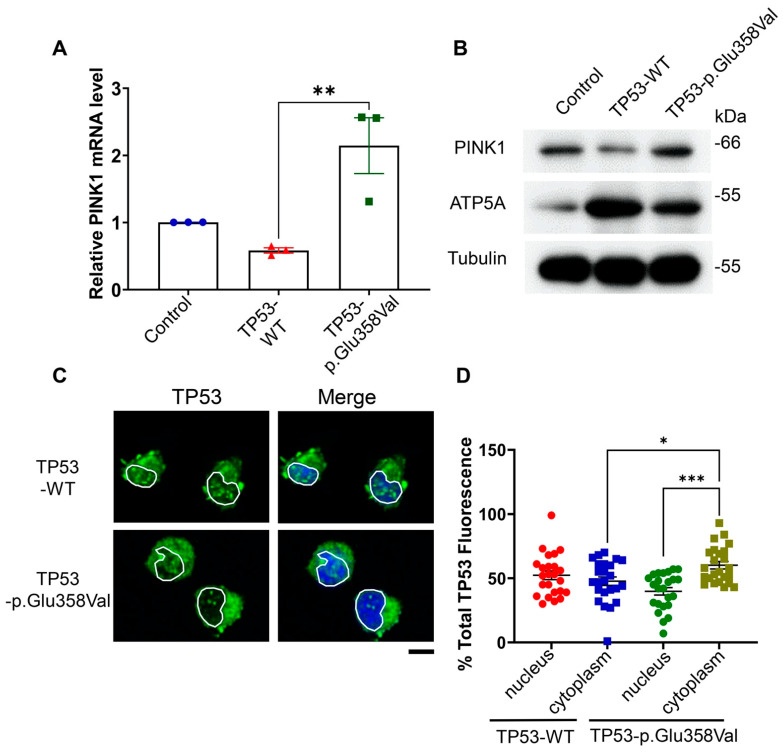
Wild-type TP53, but not TP53-p.Glu358Val, suppresses mitophagy by reducing PINK1 levels. (**A**) PINK1 mRNA levels. (**B**) Immunoblots of PINK1, ATP5A and tubulin protein levels. (**C**) Subcellular localization of TP53 in H2172 cells. DAPI is used to indicate the position of nucleus. Scale bar = 10 µm. (**D**) Quantification of TP53 fluorescence intensity in nucleus vs. cytoplasm. * *p* < 0.05, ** *p* < 0.01, *** *p* < 0.001.

A recent study demonstrates that the abrogation of TP53 activates PINK1-mediated mitophagy. PINK1, a sensor of mitochondrial damages, is required for mitophagy [28]. To test the possibility that TP53-p.Glu358Val induces mitophagy, we examined the PINK1 expression in H2172 cells expressing wild-type TP53 or TP53-p.Glu358Val. We found that the expression of wild-type TP53 in H2172 reduces both mRNA and protein levels of PINK1 compared to H2172 control cells (Figure 6A,B). Accordingly, the mitochondrial protein ATP5A is dramatically increased in H2172 expressing wild-type TP53 (Figure 6B). Consistent with previous study, these results suggest that TP53 can suppress mitophagy by blocking PINK1 expression. In contrast, TP53-p.Glu358Val expression increased PINK1 mRNA and reduced ATP5A protein levels compared to wild-type TP53 (Figure 6A,B), suggesting that, unlike wild-type TP53, TP53-p.Glu358Val fails to suppress mitophagy. Furthermore, we found that the TP53-p.Glu358Val mutant was more concentrated in the cytoplasm of H2172 cells, whereas wild-type TP53 localized at both cytoplasm and nucleus evenly (Figure 6C,D). These results suggest that TP53-p.Glu358Val mutation induces the cytoplasmic translocation of p53, leading to the activation of PINK1 transcription and mitophagy.

## 4. Discussion

Lung cancer is the highest cause of mortality worldwide. In this study, the mutation profile in a cohort of Chinese lung cancer patients led us to identify a novel mis-sense mutation of TP53, TP53-p.Glu358Val. Our results show that TP53-p.Glu358Val is a loss-of-function mutation that increases lung cancer cell growth and tumor sizes through mitophagy. We found that TP53-p.Glu358Val mutation causes the cytoplasmic translocation of TP53, leading to increases in PINK1 transcription and mitophagy. Importantly, the chemical inhibition of autophagy/mitophagy selectively limits lung cancer cell growth driven by TP53-p.Glu358Val mutation, suggesting that autophagy/mitophagy is a druggable target for lung cancers with a loss-of-function mutation of TP53 (Figure S3).

### 4.1. TP53-p.Glu358Val Mutation in Human Diseases

Besides being detected in lung cancer patients in this study, the TP53-p.Glu358Val mutation has been reported in multiple cancer types, such as breast cancer [31], chronic myelomonocytic leukemia [32] and adrenocortical cancer [33]. Interestingly, a recent study proposes that TP53-p.Glu358Val mutation exists in the germline and appears to be more specific to Asian patients [34]. The previous in silico and in vitro data predicted that TP53-p.Glu358Val mutation relates to cancer but is not pathogenic [34,35]. However, our findings strongly suggest that this *TP53* variant is clinically associated with lung cancer and contributes to oncogenic properties in lung tumor cells. One potential cause of this discrepancy may be due to tissue specificities. Future work is needed to test the effects of TP53-p.Glu358Val mutation on different tissues/cancers.

### 4.2. TP53-Dependent Regulation of Autophagy and Mitophagy

Multiple studies point to a complex regulation of TP53 on autophagy that has vast implications for the tumorigenesis process. TP53 has been shown to promote autophagy through the direct transcriptional activation of autophagy-related genes or the inactivation of mTORC1 signaling [36]. However, a recent study suggests that nuclear TP53 can suppress a selective form of autophagy, mitophagy, via the down-regulation of PINK1 expression [28]. Mitophagy is an evolutionarily conserved cellular process that selectively recycles damaged mitochondria and plays multiple roles in tumorigenesis [37]. The discrepancy suggests that nuclear TP53 may have critical roles in the PINK1-dependent control of “selective” mitophagy, besides regulating “non-selective” macro-autophagy. Similarly, in our study, we found that the cells expressing TP53-p.Glu358Val exhibit higher autophagy activities, revealed by LC3-II levels and puncta numbers, compared to cells expressing wild-type TP53. We further identified the TP53-p.Glu358Val-induced autophagy to be selective mitophagy as TP53-p.Glu358Val increases PINK1 levels and reduces mitochondrial proteins. Since mitophagy is required for cancer cell survival and tumor-promoting functions, its inhibition may serve as a prominent mechanism for anticancer therapy [37].

### 4.3. Subcellular Localization of TP53 Regulates Its Functions

It has been reported that the functions of TP53 depend on its subcellular localization, mutation status and the level of stress [38]. The TP53-p.Glu358Val mutation site lies between the nuclear export signal (NES spanning amino acids 340–351) and a nuclear localization signal (NLS spanning amino acids 369–375) [39]. Thus, it is possible that TP53-p.Glu358Val mutation alters the subcellular localization of TP53 and affects its functions. Indeed, we found that TP53-p.Glu358Val mutant proteins are enriched in the cytoplasm compared to wild-type TP53, which was distributed evenly in the nucleus and cytoplasm in our study. This phenomenon explains the increased mitophagy in H2172 cells expressing TP53-p.Glu358Val, but not wild-type TP53. The cytoplasmic localization of TP53-p.Glu358Val fails to target promoter regions of PINK1, thus leading to an increase in PINK1 expression and mitophagy. Our qPCR and immunoblotting assays both revealed that the PINK1 level is induced and that mitochondrial proteins are reduced in TP53-p.Glu358Val-expressing cells. Our findings highlight the regulation of mitophagy by the diverse localization of TP53.

### 4.4. Autophagy/Mitophagy as Druggable Targets for Lung Cancers with Loss of Function of TP53

Autophagy/mitophagy can function as a tumor suppression mechanism but can also enable tumor cell survival in environmental or pharmacologically induced stresses [22,37]. In our study, the treatment of an autophagy/mitophagy inhibitor, 3-MA, can selectively decrease the number of lung cancer cells with TP53 loss of functions. We did not observe significant cell deaths in lung cancer cells treated with 3-MA (data not shown), suggesting that the effects of 3-MA on lung cancers mainly reduce cell proliferation. It will be interesting to test other drugs targeting autophagy and mitophagy and identify inhibitors with higher efficacies. On the other hand, other potential cancer therapies targeting mutant TP53, such as MDM2 inhibitors, zinc metallochaperone-1 (ZMC1) or bispecific antibodies, have shown some efficacies on anticancer treatment [40]. It will be of interest to test whether the cotreatment of these TP53 inhibitors with 3-MA could further increase the anti-cancer efficacies.

## Figures and Tables

**Table 1:** Clinicopathological features of the cohort.

Clinicopathological Features of the Cohort	*n* = 314 n (%)
Age (median, range)	65, 29–94
Gender	
Female	112 (35.67%)
Male	202 (64.33%)
Clinical stage	
I	6 (1.91%)
II	6 (1.91%)
III	17 (5.41%)
IV	271 (86.31%)
NA	14 (4.46%)
T stage	
1	13 (4.14%)
2	22 (7.01%)
3	16 (5.09%)
4	48 (15.29%)
NA	215 (68.47%)
N stage	
0	13 (4.14%)
1	4 (1.27%)
2	32 (10.19)
3	52 (16.56%)
NA	213 (67.84%)
M stage	
0	20 (6.67%)
1	78 (24.84%)
NA	216 (68.79%)

## Data Availability

The data that support the findings of our study are available from the corresponding author upon reasonable request.

## Supplementary Materials

The following supporting information can be downloaded at: https://www.mdpi.com/article/10.3390/cells11223587/s1, Figure S1: Correlation of patient demographics, stages and TNM classification according to different muta-tion classes. Figure S2: The circos plots depict the 2-hit combinations of different genes mutation according to mutation classes. Figure S3: A working model for TP53-p.Glu358Val-driven tumor-igenesis.
