# Supplementary material for: A Novel TP53 Gene Mutation Sustains Non-Small Cell Lung Cancer through Mitophagy
Source: Cells. 2022 Nov 13;11(22):3587. doi: 10.3390/cells11223587 (PMC9688643; doi:10.3390/cells11223587)
Supplement: Supplementary file 1 [file cells-11-03587-s001.zip › cells-2009255-supplementary.pdf]

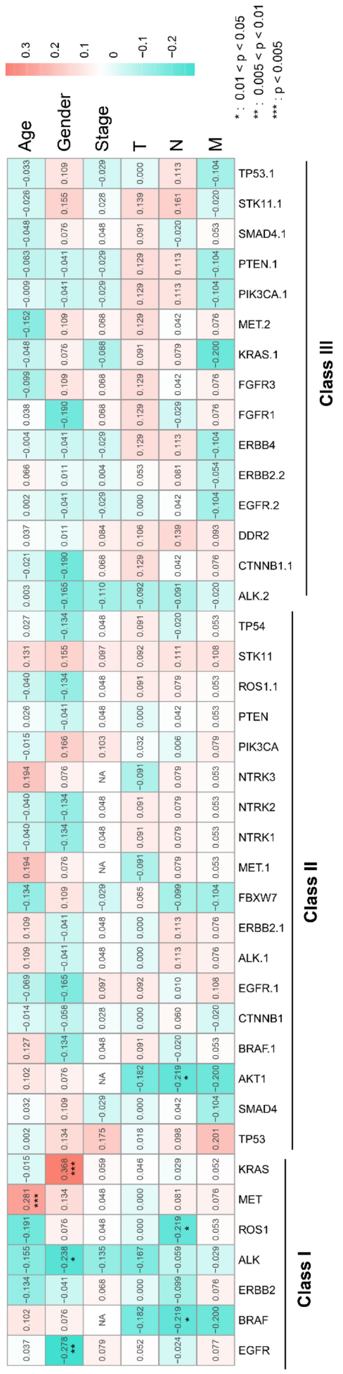

Figure S1: Correlation of patient demographics, stages and TNM classification according to different mutation classes. The intensity of redness indicates increasing positive correlation between the two factors, while greener implies the two factors are more negatively correlates with each other. *p<0.05; **p<0.01, ***p<0.005.

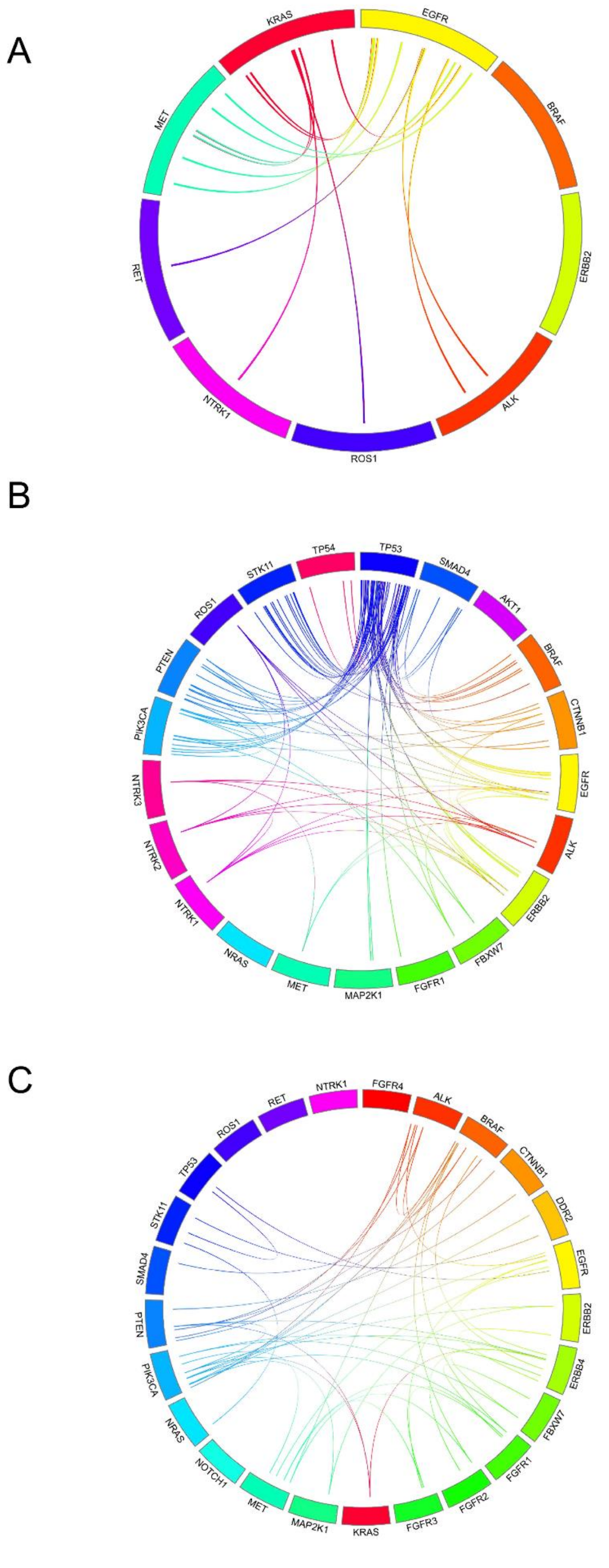

Figure S2: The circos plots depict the 2-hit combinations of different genes mutation according to mutation classes. Each line in the centre connects the two genes in a 2-hit combination. Each colour represents one gene. (A) Class I. (B) Class II. (C) Class III.

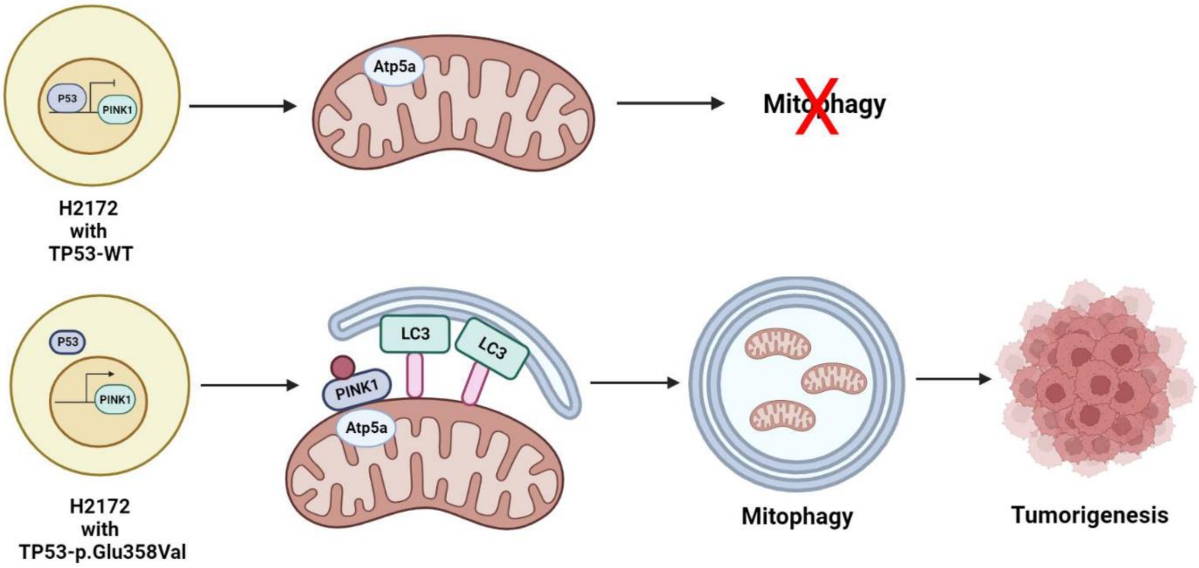

Figure S3: A working model for TP53-p.Glu358Val-driven tumorigenesis. Nuclear p53 binds to PINK1 promoter and inhibits its transcription. Cytoplasmic localization of TP53-p.Glu358Val mutants increases PINK1 expression and mitophagy. TP53-p.Glu358Val-induced mitophagy sustains lung cancer cell survival and promotes tumorigenesis.
